# Machine Learning Based Toxicity Prediction: From Chemical Structural Description to Transcriptome Analysis

**DOI:** 10.3390/ijms19082358

**Published:** 2018-08-10

**Authors:** Yunyi Wu, Guanyu Wang

**Affiliations:** Department of Biology, Guangdong Provincial Key Laboratory of Cell Microenviroment and Disease Research, Southern University of Science and Technology, Shenzhen 518055, China; wuyy3@mail.sustc.edu.cn

**Keywords:** toxicity prediction, machine learning, deep learning, transcriptome, chemical structure, molecular fingerprint, molecular fragment

## Abstract

Toxicity prediction is very important to public health. Among its many applications, toxicity prediction is essential to reduce the cost and labor of a drug’s preclinical and clinical trials, because a lot of drug evaluations (cellular, animal, and clinical) can be spared due to the predicted toxicity. In the era of Big Data and artificial intelligence, toxicity prediction can benefit from machine learning, which has been widely used in many fields such as natural language processing, speech recognition, image recognition, computational chemistry, and bioinformatics, with excellent performance. In this article, we review machine learning methods that have been applied to toxicity prediction, including deep learning, random forests, k-nearest neighbors, and support vector machines. We also discuss the input parameter to the machine learning algorithm, especially its shift from chemical structural description only to that combined with human transcriptome data analysis, which can greatly enhance prediction accuracy.

## 1. Introduction

**Figure 1:**
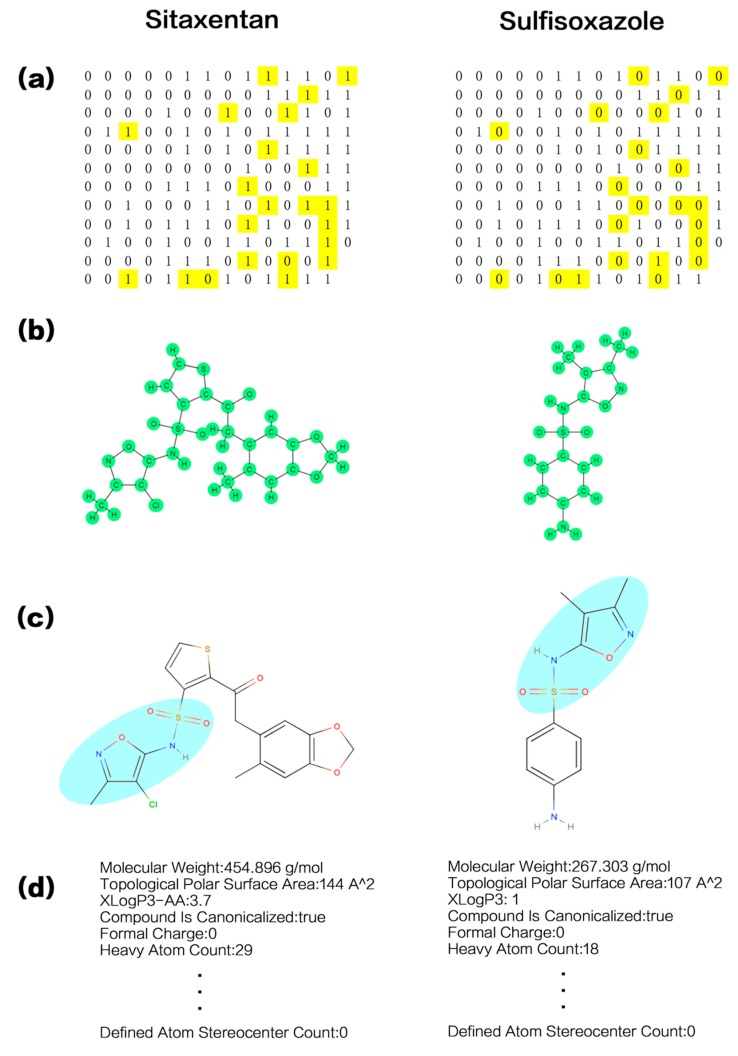
Chemical structural description of Sitaxentan and Sulfisoxazole. (**a**) The 166-bit molecular access system (MACCS) molecular fingerprints, where the different values are indicated in yellow; (**b**) The undirected graphs with atoms as nodes and bonds as edges; (**c**) The molecular structures of Sitaxentan and Sulfisoxazole, where the cyan regions are their common molecular fragment identified by CNN training; (**d**) Other chemical properties.

Toxicity evaluation is of fundamental importance in drug development and approval. It is well known that drugs must undergo clinical trials to become legal [1,2]. Unfortunately, clinical trials are always associated with certain degree of risk. It was reported that about half of the new drugs were found to be unsafe or ineffective in late human clinical trials [3]. For example, the drug Sitaxentan (Figure 1) was urgently withdrawn from global markets due to specific and irreversible hepatotoxicity in humans [4,5]. Unsafety of clinical trials highlights the importance of preclinical evaluations, which are absolutely necessary in order to prevent toxic drugs from entering into clinical trials.

The animal trial, a common method of preclinical evaluation, is of limited value. On the one hand, the trial is very expensive and laborious. On the other hand, the results offer little guidance to human toxicity reactions, due to inter-species differences and differential disease models [6,7]. For example, Sitaxentan caused no explicit liver injury in animal experiments [8], whereas the hepatotoxicity was prominent in humans [4,5]. Therefore, animal experiments cannot tell the human body’s response to new drugs and offer no risk exemption [6,9].

To reduce the expenses and uncertainties inherent of animal experiments, it is crucial to perform high-throughput computer toxicity predictions. One dominant and most developed toxicity prediction method is Quantitative Structure-Activity Relationships (QSAR) based on chemical structural parameters [10]. This method uses statistics to establish, for a drug compound, a quantitative relationship between the structural or physicochemical characteristics and its physiological activities [11]. From the relationship, one can predict the physiological activities or other properties of the compound, including toxicity. The earliest and widely used QSAR method was the Hansch approach, as proposed in 1962 [12], which assumes independence of the factors modulating the compounds’ biological activities. It relies on methods that are related to free energy and statistical methods, such as linear regression, to obtain the QSAR model [12]. The Free-Wilson method, as proposed in 1964, directly used molecular structure as a variable for regression analysis of physiological activity [13]. In the 1980s, QSAR regression analysis began its application in drug toxicity prediction [14,15,16]. At the turn of the 21st century, researchers performed toxicity prediction based on single or multiple physicochemical mechanisms [17]. For the single mechanism, linear regression analysis, multivariate analysis, and neural network models were primarily used. For the multiple mechanisms, knowledge based systems were often used besides statistical approaches. Nowadays, with the amount of data increasing explosively, it becomes more and more difficult to maintain completeness of knowledge bases; thus, knowledge-based systems are difficult to complete highly automated work with high volume of data [18]. Meanwhile, statistical approaches, such as linear regression analysis, multivariate analysis, and early shallow neural network models are difficult to extract more abstract features, and are thus difficult to predict with high accuracy. 

To address these new challenges, researchers made great efforts to improve both the prediction model (development of machine learning) and inputs to the prediction model (characterization of chemical structure descriptors). The two lines of works interacted with each other and synergistically promoted the field of computer-based toxicity prediction. They are discussed, respectively, in the following. 

## 2. Machine Learning

Machine learning is a branch of artificial intelligence that uses sophisticated algorithms to give computers the ability to learn from the data and make predictions [19]. Main algorithms of machine learning, evolved from the study of cluster analysis and pattern recognition, include artificial neural networks (ANN), decision trees, support vector machines (SVM), and Bayesian classifiers [20]. Besides cluster analysis and pattern recognition, these algorithms have been widely linked to data mining [21].

Due to merits of machine learning, such as fastness, cost-effectiveness, and high accuracy, more and more researchers use machine learning to predict toxicity [22]. Researchers have used a combination of algorithms, such as genetic algorithm (GA) [23,24], random forest (RF) [25,26,27], artificial neural network (ANN) [28,29,30], and other machine learning algorithms [31,32,33] to optimize traditional QSAR models in predicting a drug’s toxicity or other biological activities. Different machine learning methods perform differently. Factors such as datasets and computational representations can significantly affect the performance.

### 2.1. Shallow Architectures

In 1957, Rosenblatt put forward a perceptron model simulating the structure of a neuron, which can be used as a binary classifier [34]. Widrow and Hoff first used Delta rules to train the perceptron and laid the foundation for linear classifier [35]. In 1967, Cover and Hart proposed the nearest neighbor algorithm, which allows for computers to classify sample points according to spatial features [36]. In 1986, Quilan proposed the decision tree algorithm [37]. In 1995, Cortes et al. came up with SVM, the key idea of which was to find a boundary that divides two categories with the largest distance. Besides the linear classification, SVM can be applied to high dimensional nonlinear classification [38]. In 2001, Breiman gave rise to the RF algorithm [39], which is a classifier with multiple decision trees. Individual trees output their respective prediction category, which then vote to determine the final category output of the classifier [40]. It is widely used in solving multiclass problems. SVMs and RFs are both based on statistics; they thus perform well in structured and denser datasets. 

In 1986, Hinton et al. invented the back-propagating algorithm (BP) of multi-layer perceptron (MLP) with a sigmoid activation function to perform nonlinear mapping, and used ANN effectively to solve the problem of nonlinear classification and training [41]. Soon, in 1991, it was pointed out that BP with sigmoid activation function has the vanishing gradient problem and is thus difficult to follow deeper and more abstract training [42]. These ANN architectures are thus called shallow learning.

### 2.2. Deep Learning

In 2011, the ReLU (Rectified Linear Unit) activation function was first proposed [43], which solved the vanishing gradient problem inherent of the sigmoid function. This breakthrough signified the birth of deep learning [44]. Algorithms that are based on ReLU activation function have obtained compelling performance in the field of image recognition [45,46].

As an extension to ANN, deep learning has become a very successful branch of machine learning. It innovates many fields, including pattern recognition, speech recognition [47,48], natural language processing [49,50], image and video recognitions [45,51,52], and life science [53,54]. Deep learning excels when the working data are unstructured, sparse, and large. In recent years, two neural network models, recurrent neural networks (RNN) [55,56], and convolutional neural networks (CNN) [57,58], have been commonly used in deep learning. The former is more suitable for prediction or recognition of sequences, such as natural language processing [59] and time series prediction [60,61]. The latter is more suitable for the recognition of spatial arrangement features, such as the shapes in graphics and images [62].

With the increase of computer speed, the deployment of large-scale distributed clusters [63] and GPUs [64], and the emergence of numerous optimization algorithms [65], deep learning training time reduced greatly and it is now useful to both bioinformatics [66,67] and chemoinformatics [68,69].

## 3. Chemical Structure Descriptors

Information for toxicity prediction is primarily from the drug compound’s chemical structure. To be understood by computers, the chemical structures need to be represented by numbers or characters, the so-called chemical descriptors. Only after chemical structures are converted into descriptors, can the computers efficiently process a large amount of structures, via the computers’ high-throughput data processing capacity. 

Cammarata and Menon first proposed a molecule-based pattern structure, and established an 8-bit digital chemical descriptor [70,71]. Later, researchers added first-order molecular connectivity values to the existing descriptor indices, for the structural classification of compounds [72]. In addition, a lot of researchers have applied quantum chemistry in order to calculate molecular descriptors (e.g., [73]). By 2000, atoms and bond multiplicity were added to describe the structural parameters of the topology; molecular hydrological, steric, or electronic descriptors were added to explore the relationship between biological activity and chemical structure as well [74]. Around 2001, researchers began to take the three-dimensional (3D) structure of molecules into account to establish 3D-QSAR chemical descriptors [75,76]; some went a step further to generate four-dimensional (4D)-QSAR chemical descriptors by adding molecular dynamics (MD) trajectories and topological information [77].

The descriptor types vary from simple features, like atomic counts or molecular weights to structural features [78]. Different combinations of chemical descriptors and machine learning models might perform differently.

### 3.1. Traditional Chemical Descriptors

Traditional chemical descriptors are those that are calculated mainly based on molecular structure-derived information, like atomic types, atomic charges, or atomic distances. Table 1 presents the main types of traditional chemical descriptors that are categorized by the calculation parameters [79]. Among them, molecular fingerprints are the most widely used, which are in the form of an array of numbers. They use information, such as atomic attributes, atomic environments, bond properties, and bond position to encode chemical structures [80]. Among them, the 166-bit molecular access system (MACCS) is a typical one (Figure 1a). Each of the 166 bits encodes a specific structural characteristic, such as: whether or not the number of methyl groups in the molecule is greater than 1? whether or not the molecule is aromatic [81]?

The importance of molecular fingerprint is easily seen: for those active substances whose functional groups happen to locate at “ortho” or “meta” positions, their toxicity can usually be predicted correctly with MACCS or extended connectivity fingerprint (ECFP) [82]. Autoencoder and convolution based methods are used to predict the chemical properties where chemicals are signified by vectors of fixed length, just like MACCS [68]. In experiments involving combinations of molecular fingerprint and machine learning, Pubchem-SVM and MACCS-RF are the two best combinations. The merits of SVM and RF are apparent. SVM performs the best among many machine learning models, including SVM, RF, k-nearest neighbor (k-NN), and naive Bayes [83]. On the other hand, RF is structured by many decision trees, which are trees with “yes” and “no” as their leaves. Since “yes” and “no” are represented by 1 and 0, respectively, RFs correspond naturally to molecular fingerprints or other chemical descriptors, which consist of many binary digits (0 or 1).

### 3.2. Deep-Minded Chemical Descriptor

Molecular fingerprints encode chemical structures in great detail (every atom or bond), which may sometimes be unnecessary or even disadvantageous (complicated and inefficient). To obtain a coarse grained, but more deep-mined model, researchers characterized molecules by deep learning architectures, such as RNN and CNN. 

One learning method is based on the two-dimensional planar molecular structure, whereby the entire molecule is converted into an undirected graph (Figure 1b). With atoms as nodes and bonds as edges, each node is sequentially traversed [68,84,85]. This would permit an understanding of the relationship between structure and reactivity [86]. Being sensitive to time sequence or succession, RNN and its variant long short-term memory (LSTM) are used to construct this kind of molecular fragments [84,85]. 

Two-dimensional fragments can be constructed directly from the molecule (Figure 1c), without sequentially traversing every atom in the undirected graph by RNN. CNN classifies the molecules into molecular fragments, which are chemical substructures that are not naturally classified according to the functional groups, but they are adjusted constantly by the “learning” machine. The final molecular fragments should be more interpretable and readable [87]. Using CNN to automatically construct abstract chemical fragments, the deep learning model showed very high performance in toxicity prediction based on high-throughput data, with an average area under the curve (AUC) of 0.846 [88]. The AUC value is the probability, according to the result of the current algorithm, that the positive sample is ranked before the negative sample when both samples are randomly picked by the algorithm [89]. The greater the AUC value, the more likely the current classification algorithm placing the positive sample before the negative one, and the better the classification.

Figure 1c gives an example of CNN identifying the same substructure (colored in cyan) from two different molecules. It can identify even smaller substructures. After extensive data training, CNN can identify those substructure or molecular fragments that might make a molecule toxic. When working on new test sets, CNN usually predicts with high accuracy [88].

### 3.3. Chemical Properties

Being determined by molecular structures, chemical properties (molecular weight, degradation rate, solubility coefficient in different solvents, molar index, permeability, etc.) can also be used for classification and prediction (e.g., Figure 1d) [90,91]. The use of molecular descriptor parameters that are derived from electronegativity and covalent radii of forming atoms and interatomic distances can also improve prediction by ANNs [92]. Molecular fingerprints that based on both simple molecular properties and characteristics derived from two-dimensional molecular structures, such as measurements of lipophilicity (LogP and LogD) and topological polar surface area (TPSA), were combined with a variety of machine learning models (e.g., RF, SVM, k-NN) for toxicity prediction and classification. By comparing their performances, it was found that RF usually outperformed [91]. Using the k-NN algorithm, Chavan et al. even tried to predict the chronic toxicity of chemical substances by combining acute toxicity information with molecular fingerprints such as MACCS and CDK [93]. These studies demonstrated that chemical properties can help improve accuracy of toxicity prediction. 

### 3.4. Examples of Chemical Structural Description

Sitaxentan is a drug to treat pulmonary arterial hypertension (PAH) and Sulfisoxazole is a sulfonamide antimicrobial with some hepatotoxicity implications [94]. Their structural descriptions are presented in Figure 1. The two drugs have 22/166 different places and 144/166 identical places in the MACCS molecular fingerprint (Figure 1a). The explicit binary structure of the MACCS molecular fingerprint is well-suited to the structural characteristics of the decision tree algorithm; thus, RF outperformed other machine learning models when dealing with MACCS. Figure 1b displays the undirected graphs of Sitaxentan and Sulfisoxazole, with atoms as nodes and bonds as edges. Every node corresponds to a vector whose terminal point is just the node. The vector can be constructed from the undirected graph by determining the paths of all the other nodes to the terminal point. Finally, all of the vectors are added to form the molecular structure vector of the corresponding molecule [68]. In Figure 1c, the cyan region indicates the same substructure of the two molecules that are identified by CNN. Figure 1d gives the other chemical properties of these two molecules.

## 4. Chemical Structure Based Toxicity Prediction by Machine Learning

After using computer-readable and interpretable methods to represent the molecular structure, a machine learning model is trained to predict toxicity.

### 4.1. Data Collection

**Figure 2:**
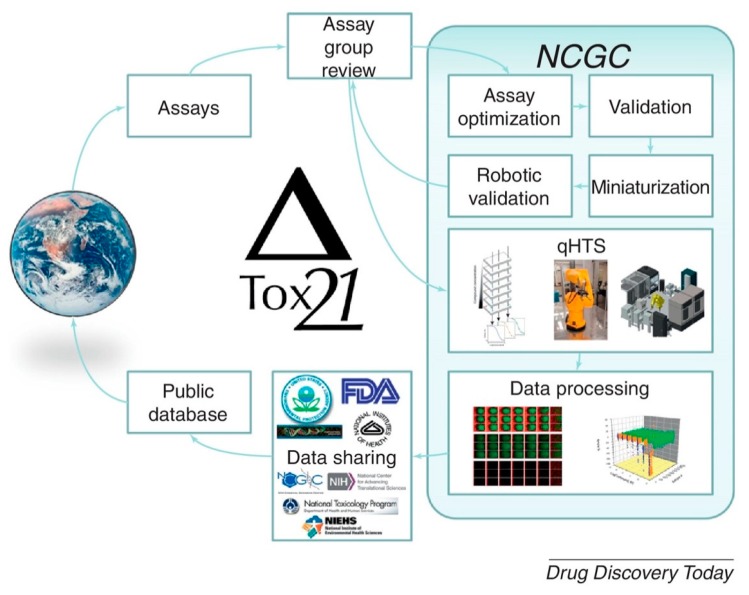
Tox21 screening workflow in drug discovery (qHTS: quantitative high-throughput screening; NCGC: NIH Chemical Genomics Center) [105].

Accuracy of toxicity prediction depends on the amount of data being collected. During the past years, extensive data collections have resulted in some mainstream toxicity databases (Table 2). Toxicology data network (TOXNET), which was created in 1985, is among the world’s largest collection of toxicology databases. The first database that was added to the network was the Hazardous Substances Data Bank (HSDB), which contains acute-toxicity information [95,96]. Toxicity ForeCaster (ToxCast) is also a widely used high-throughput toxicity database. It is a part of the Toxicology in the 21st Century (Tox21), whose screening workflow is represented in Figure 2. Tox21 contains both acute and chronic toxicity information.

### 4.2. Performance

The prediction model, which was obtained by combining machine learning and the molecular descriptors, is similar to QSAR, which has long been used to study the quantitative relationship between molecular structure and biological activity [106]. The latter includes toxicity and environmental behavior of chemicals, which makes QSAR one conventional method to predict toxicity [107,108]. Here, we mainly discuss QSAR studies that are based on the two-dimensional structure of chemical molecules combined with biological activity parameters. In the earliest days, researchers used simple pattern recognition methods, such as k-NN, to classify and predict compound toxicity. But, simple pattern recognition is difficult to process asymmetric data, in which positive samples are far less than negative ones, or vice versa [109]. Asymmetric data are ubiquitous in the toxicity database, because non-toxic compounds are not specifically labeled in the database. Fortunately, ANNs and algorithms of the decision tree class, including random forests, can well classify and predict asymmetric data or imbalanced data showing a strong generalization ability [110,111,112]. For example, with the loss function improving, deep neural networks (DNNs) exhibited excellent performance for classifying even extremely imbalanced data [113].

With molecular fingerprints ECFP6, FP2, MACCS combined with ANN models, the two-dimensional QSAR virtual screening can achieve an average r test value (which measures regression fitness) of 0.75 [114,115]. Deep learning multi-task neural networks worked so well that the AUC value for toxicity QSAR prediction of NIH/3T3 cells (mouse embryonic fibroblast) can reach 0.9, which is slightly higher than the AUC of 0.87 in random forests, in which molecular fingerprints as input of the model [116]. Besides ANNs, RFs have also been successfully applied to QSAR predictions. Using a molecular fingerprint or a simplex representation of molecular structure to store chemical molecular structure information, such as atom type and other physical-chemical characteristics of an atom, RF was validated on the QSAR external test set [25,117]. In addition, Wu et al. recently improved traditional molecular descriptors using element specific persistent homology (ESPH) and auxiliary descriptors, where ESPH includes topological information from intermolecular interactions and homology analysis on each component of molecules. On this basis, they performed RF, Gradient Boosting Decision Tree, single-task deep learning, multi-task deep learning, multi-task deep learning methods, and achieved the highest degree of fitness and accuracy [118].

Table 3 presents the AUC values of different machine learning models combined with different molecular descriptors. One sees that traditional machine learning methods such as SVM and RF have higher AUC values than deep learning algorithms. The reason might be that currently available toxicity datasets are not sufficiently large to support deep learning algorithms to further improve their accuracy. Otherwise, the accuracy of deep learning would increase markedly due to semi-supervised learning characteristics.

## 5. Acute (Immediate) Toxicity Prediction

Toxicity can be divided into acute toxicity and chronic toxicity. The latter includes toxicity to reproduction, mutagenicity, and carcinogenicity [121]. Acute toxicity is usually measured by LD50 (Lethal Dose 50) for drug testing and LC50 (Lethal Concentration 50) for environmental sciences [122]. In 1997, Gute and Basak used the simplest linear regression to predict acute aquatic toxicity [123]. In 2000, Basak et al. used ANN to predict LC50 of benzene derivatives [124]. After the development of machine learning, in 2011, Lu et al. used k-NN combined linear regression model to predict acute oral toxicity in rats and achieved a R square value of 0.712, for which they utilized the local chemical structure that was represented by molecular fingerprints [31]. Martin et al. used the global hierarchical clustering method to predict acute toxicity of pesticides and obtained better results than linear regression [125]. 

**Figure 3:**
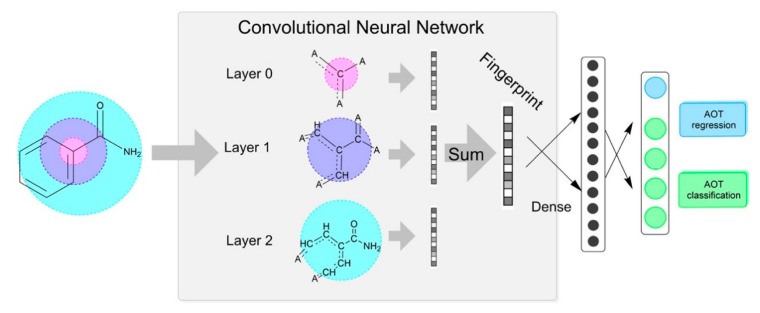
An acute oral toxicity prediction. The prediction starts from a chemical molecular structure in the simplified molecular-input line-entry system (SMILES) format, as an input to the MEG-CNN, where the pink, purple, and cyan circles represent the first, second, and third iterations, respectively. During each iteration, the chemical structure is processed by the convolutional kernel according to the atom degree to obtain the corresponding pre-fingerprint. All of the pre-fingerprints are integrated to generate the fingerprint, which was further processed to generate the deep-mined fingerprint. The deep-minded fingerprint was then tested by the regression model (the blue circle) and the multiclass/multitask models (the green circles) [127].

Recently, Liu et al. compared performance of shallow architectures, such as RF and k-NN, with DNN in acute toxicity prediction based on extremely unbalanced datasets. For the sake of fairness, they used the chemical descriptor of ECFP uniformly. It was found that RF and DNN performed better on the global dataset, while k-NN performed better on the unbalanced acute toxicity datasets. This result also highlights the importance of neighbor information in acute toxicity prediction [126]. In order to adapt the chemical descriptor to the prediction model, Xu et al. used an enhanced molecular graph encoding convolutional neural networks (MGE-CNN) (the gray box in Figure 3) to process the standard molecular structure data, and finally obtained the fingerprint. The fingerprint was further mined both forwardly and backwardly, which yielded the deep-minded fingerprint (the array of black dots in Figure 3). The deep-minded fingerprint was then tested by the regression model (the blue circle) and the multiclass/multitask models (the green circles), which yielded a classification accuracy up to 95.0% and a regression R square value of 0.861 [127].

## 6. Chronic (Delayed) Toxicity Prediction

### 6.1. Prediction Based on Chemical Structure

When compared with acute toxicity, chronic toxicity is more latent and hard to discover. Chavan et al. classified the LD50 values of compounds using k-NN. Based on the classification, they predicted the LOEL (lowest observed effect level), which was then used to measure chronic toxicity. The R square value of the test set was only 0.54, however [93]. In 2017, Li et al. used machine learning models, such as RF, SVM, and k-NN to predict the oral LOAEL (lowest observed adverse effect level) of rats. The method k-NN obtained the best performance, yielding AUC values up to 0.814 [128].

### 6.2. Prediction with Cellular Transcriptome Information

Chemical structure based toxicity prediction is only the first step of drug evaluation. The subsequent steps include cell, animal, and clinical toxicity tests. Because drugs are designed for humans, toxicity testing on human cells is both clinically relevant and cost effective. Whole genome expression, or transcriptome expression, reflects the state changes of a cell, either in vivo or in vitro. For example, if a cell has a high expression of a proto-oncogene, then the chance is high of the cell’s carcinogenesis. Therefore, machines should fully exploit gene expression data for feature selection and classification in drug trials [129]. Deep-sequencing RNA-Seq technology has led to an unrivaled explosion in the amount of data, which would help researchers to gain a deeper understanding of biological mechanisms (e.g., changes of cellular signaling pathways) of toxic compounds, such as Benzo[a]pyrene. This would, in turn, help researchers to better characterize harmful effects that are caused by chemicals [130].

These technical developments make the following strategies practical. One can induce changes of whole genome expression of cultured human cells of a specific type by adding a test drug to the culture. By analyzing changes in the transcriptome, toxicity of the drug to the cell type, and to the corresponding organ, can be predicted [131]. Schwartz et al. used both toxic and non-toxic compounds to treat 3D-cultured human pluripotent stem cell-derived neural cells, then used RNA-Seq to determine the whole genome expression profile, and then used SVM to classify the chemicals according to their toxicity. The scheme gained an average AUC value of 0.91 [132]. Yamane et al. used chemicals to treat human embryonic stem cells and analyzed their transcriptomes. By classifying the chemicals into different categories, such as neurotoxins, genotoxic carcinogens, and non-genotoxic carcinogens, and by analyzing gene interaction networks, they gained much richer information, which greatly improved the accuracy of toxicity prediction and even allowed for them to predict the delayed chemical toxicity with SVM [133]. What underlay their success was the fact that delayed toxicity is associated with changes in gene expression, which can, in turn, affect the expression of downstream genes [134,135]. Although the number of affected genes is small at the induction, much greater gene expression changes will occur 24 h after induction [136]. Therefore, the accurate prediction of late-onset chemical toxicity might be ascribed to the analysis of gene interaction networks: alterations that are caused by a compound propagate through gene-gene interactions; and, the chain reactions finally lead to genome instability and cytotoxicity. Because gene expression is not immediate, toxicity onset is often delayed and it is difficult to detect immediately after the induction. Following the same logic, the degree of toxicity would positively correlate with the degree of connectivity of the genetic network, because the number of affected genes would increase explosively as the complexity of the network increases [137,138].

Based on a large-scale dataset of gene expression, and by using drugs’ chemical structure as the input and the altered gene expression as the output, Liu et al. established a variable-nearest neighbor model to predict the QSAR between chemical structures and gene expression profiles, and obtained an AUC value of more than 0.7 [139].

## 7. An in Silico Platform of Deep Learning Based Toxicity Prediction

**Figure 4:**
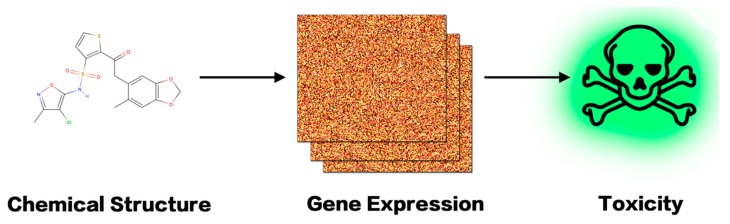
Toxicity prediction with gene expression data.

On the basis of the above researches, we are establishing a pertinent system encompassing all of the major aspects of toxicity prediction: chemical structure, gene expression, deep learning, etc. Besides immediate toxicity prediction, delayed toxicity can also be predicted (Figure 4). In this system, drug molecular structures are represented by chemical fragments learned by CNN [88]. Gene expression data are mainly obtained by splicing gene embedding identified by RNA-Seq.

### 7.1. Collection of Gene Expression Data

Table 4 represents the databases we are using to gain gene expression data after drug treatment to the cells. Among the databases, CMap is the most popular one to analyze the relationship between transcriptome data and drugs [140].

### 7.2. Representation of Gene Expression Data

Each of these human gene embeddings can be represented by a 300-dimensional gene vector trained from 984 datasets of the GEO database based on gene co-expression patterns [150]. This vector representation reflects gene functions indirectly. Besides this co-expression based gene embedding, there are other methods for vector representation of genes. One method is similar to word2vec used in natural language processing [151,152]. The method word2vec converts words into vectors that are computer understandable by using shallow neural networks with a large amount of neurons. In another method, vectors are constructed based on a similarity of different gene annotations in Gene Ontology, which allows for the quantification of similarities between genes [153]. This representation directly reflects gene functions and indirectly reflects gene interactions. Besides the use of gene vectors, the dimension of RNA-Seq data can be reduced by techniques, such as Stacked Denoising Autoencoder (SDAE), which allows for the discovery of gene interaction patterns [154] and specific gene expression patterns [155] by extracting features from RNA-Seq data by a supervised learning classification model. By scoring pathway activation and regarding “landmark genes” as new features to perform dimensionality reduction, Aliper et al. combined processed gene expression data with DNNs to identify the pharmacological properties of multiple drugs under different biological systems and conditions [156].

**Figure 5:**
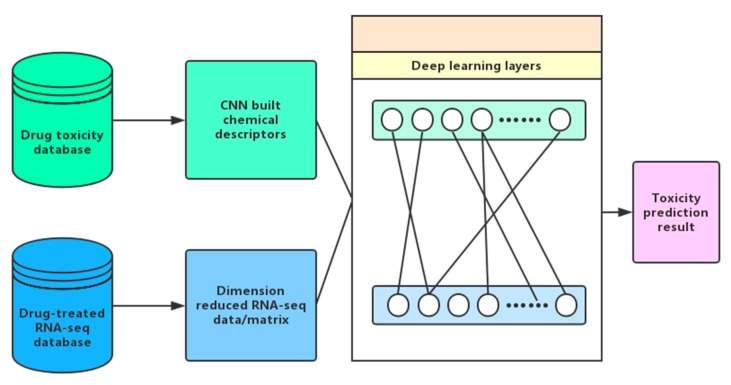
Toxicity prediction with RNA-seq data.

With gene expression data at hand and with chemical structures digitalized, one can use the system to find deeper and intrinsic links between the two through machine learning models (Figure 5), by either establishing the association with chemical structures as input and gene expressions as output (from structure to effect), or vice versa (from effect to structure). The former can help with QSAR prediction, including toxicity, while the latter can help with the design of inducing drugs based on the desired changes of gene expression pattern.

### 7.3. Toxicity Prediction

Incorporating genetic information would render more accurate toxicity prediction and QSAR construction [133]. The fundamental reason is that changes in gene expression provide biological information, which is much richer and more complex than the simple molecular structure and chemical properties. Furthermore, the biological information is not only at the molecular level, involving only a single pair of drug-protein interaction, but also at the systems level with a drug targeting the whole gene interaction network, affecting the whole cell and even the whole organism.

One can not only distinguish between toxic and non-toxic, but also perform classified toxicity prediction (neurotoxins, carcinogens, etc.). For example, Gayvert et al. performed classified toxicity prediction on FDA-approved drugs and drugs that had failed to pass toxicity-tests, with the RF supervised learning algorithm. The learning was from multiple sources: chemical structure characterizations, the median value of the expression of the drug targeted genes from the transcriptome of various tissues, the frequency or possibility of functional mutations (i.e., drug induced gene mutations that lead to loss of function). They finally obtained an AUC value of about 0.8263 [157]. Calculation of median expression of drug target genes is useful, but they may ignore tissue specificity and differential toxic reactions. For example, a toxic drug may induce high expression of a particular gene only in the liver, but not in the other organs or tissues. The median value of the gene expression, being based on the whole body measurement, is thus very low and cannot reflect the drug’s toxicity specific to the liver. In this event, the use of tissue transcriptome data might be more specific and can help to extract more relevant features.

When compared with the random forest approach, the deep learning approach can handle higher throughput and larger amounts of data, and be capable to deal with higher-level and more abstract features, resulting in a better performance after subsequent data accumulation.

## 8. Summary

The 21st century has witnessed the rapid development of artificial intelligence, including machine learning. This rapid development is partly stimulated by its many important applications, one of which is drug toxicity prediction in silico [88,127,158]. Together with “Big Data” science [159], machine learning techniques may provide much more information about toxicity than ever before.

In this article, we have reviewed machine learning methods that have been applied to toxicity prediction. We have also discussed the input parameter to the machine learning algorithm, especially its shift from chemical structural description only to that combined with human transcriptome data analysis, which can greatly enhance prediction accuracy.

The merits of machine learning based toxicity prediction are summarized, as follows. Firstly, many harmful and risky animal or clinical trials can be spared, due to toxicity predicted by computers. Secondly, in silico prediction is risk-free, low-costly, and of high throughput. Thirdly, because human transcriptome data are often used, the prediction is essentially based on system-level complexities; the method is thus more global than those studying single protein related toxicity. Finally, due to its capacity of extracting complex and abstract features in pharmacology and bioinformatics applications [160], machine learning may eventually become completely in silico, as the data continue to expand and the accuracy continues to improve.

## Figures and Tables

**Table 1:** The main types of traditional chemical descriptors [79].

Descriptor Type	Descriptor Name	Description
Fingerprint-based	ECFP4	atom type, extended connectivity fingerprint, maximum distance = 4
	FCFP4	functional-class-based, extended connectivity fingerprint, maximum distance = 4
	MACCS	166 predefined MDL keys (public set)
Connectivity-matrix-based	BCUT	atomic charges, polarizabilities, H-bond donor and acceptor abilities, and H-bonding modes of intermolecular interaction
Shape-based	rapid overlay of chemical structures (ROCS), combo Tanimoto (shape and electrostatic score)	shape-based molecular similarity method; molecules are described by smooth Gaussian function and pharmacophore points
	PMI	normalized principal moment-of-inertia ratios
Pharmacophore-based	GpiDAPH3	graph-based 3-point pharmacophore, eight atom types computed from three atom properties (in pi system, donor, acceptor)
	TGD	typed graph distances, atom typing (donor, acceptor, polar, anion, cation, hydrophobe)
	TAD	typed atom distances, atom typing (donor, acceptor, polar, anion, cation, hydrophobe)
Bioactivity-based	Bayes affinity fingerprints	bioactivity model based on multicategory Bayes classifier trained on data from ChEMBL v. 14
Physicochemical-property-based	prop2D	physicochemical properties (such as molecular weight, atom counts, partial charges, hydrophobicity etc.)

**Table 2:** The mainstream data resources of toxicity chemicals.

Database	Database Description	Online Websites	Reference
TOXNET	A collection of toxicity databases.	https://toxnet.nlm.nih.gov/	[97]
ToxCast	High-throughput toxicity data on thousands of chemicals.	https://www.epa.gov/chemical-research/toxicity-forecaster-toxcasttm-data	[98]
Tox21	(1) Chemical Effects in Biological Systems; (2) Individual data and summaries from National Toxicology Program studies; (3) The growth, survival, pathology and other toxicology data.	https://ntp.niehs.nih.gov/results/dbsearch/index.html	[99,100]
PubChem	(1) Chemical structures; (2) Identifiers; (3) Chemical and physical properties; (4) Biological activities; (5) Toxicity data (6) Patents and health, safety and so on.	https://pubchem.ncbi.nlm.nih.gov/	[94]
DrugBank	Detailed drug data and corresponding drug target information.	https://www.drugbank.ca/	[101]
ToxBank Data Warehouse	Data for systemic toxicity.	http://www.toxbank.net/data-warehouse	[102]
ECOTOX	Single chemical environmental toxicity data on aquatic life, terrestrial plants and wildlife.	https://cfpub.epa.gov/ecotox/index.html	[103]
SuperToxic	Toxic compound data from literature and web sources.	http://bioinformatics.charite.de/supertoxic/	[104]

**Table 3:** Comparison of area under the curve (AUC) scores among different combinations of molecular descriptors and machine learning models.

	Molecular Descriptor	Model	AUC	Reference
Shallow architectures	Dragon descriptors (2489 descriptors)	RF	0.81	[119]
	Pubchem keys	SVM	0.948	[83]
	MACCS fingerprints	RF	0.947	[83]
Deep learning	Molecular fragments learned by CNN	DNN	0.837	[88]
	Unidirectional graph learned by CNN	Graph CNN	0.867	[120]
	LSTM graph	One-shot learning	0.84	[84]

**Table 4:** Databases of drug induced gene expression.

Database	Description	Websites	References
GEO database	Gene expression data of drug-treated samples in subsets.	https://www.ncbi.nlm.nih.gov/geo/	[141,142]
Connectivity Map (CMap)	(1) Genome-wide transcriptional expression data from cultured human cells treated with bioactive small molecules; (2) Simple pattern-matching of functional connections between drugs, genes and diseases through the transitory feature of common gene-expression changes.	https://portals.broadinstitute.org/cmap/	[140]
DSigDB	(1) Drug and small molecule-related genes based on quantitative inhibition; (2) Drug-induced gene expression changes data.	http://tanlab.ucdenver.edu/DSigDB	[143]
LINCS Canvas Browser (LCB)	(1) Experiment data about the landmark gene expression changes in response to a drug; (2) Both gene expression records before and after drug application.	http://www.maayanlab.net/LINCS/LCB	[144]
Therapeutic target database (TTD)	(1) Drug resistance mutations in drug-target genes; (2) Drug resistance mutations in regulatory genes; (3) Differential expression profiles of drug-targets in the disease-relevant drug-targeted tissues of different diseases; (4) Expression profiles of drug-targets in the non-targeted tissues of healthy individuals; (5) Target combinations of different drugs.	http://bidd.nus.edu.sg/group/ttd/ttd.asp	[145]
Comparative Toxicogenomics Database (CTD)	(1) Cross-species chemical-gene/protein interactions data; (2) Chemical- and gene-disease relationships.	http://ctdbase.org/	[146]
Drug-Path	Drug-induced pathways.	http://www.cuilab.cn/drugpath	[147]
CancerDR	(1) Anticancer drugs and their effectiveness against cancer cell lines; (2) Drug target gene information like function, structure, and gene sequences in respective cancer cell lines.	http://crdd.osdd.net/raghava/cancerdr/	[148]
KEGG DRUG	(1) Chemical structures and/or chemical components; (2) The interaction network with target molecules, metabolizing enzymes, and other drugs; (3) The chemical structure transformation network in the history of drug development.	https://www.genome.jp/kegg/drug/	[149]
